# Accessing Nystatin through Mariculture

**DOI:** 10.3390/molecules26247649

**Published:** 2021-12-17

**Authors:** James J. La Clair

**Affiliations:** Xenobe Research Institute, P.O. Box 3052, San Diego, CA 92163-1052, USA; i@xenobe.org

**Keywords:** natural product production, mariculture, drug development, marine biotechnology

## Abstract

Understanding our oceans and their marine ecosystems has enabled the development of sustainable systems for mariculture. While the bulk of studies to date have focused on the production of food, its remarkable expanse has inspired the translation of other markets towards aquatic environments. This manuscript outlines an approach to pharmaceutical mariculture, by demonstrating a benchmark for future prototyping. Here, design, field evaluation and natural product chemistry are united to successfully produce nystatin at sea. This study begins by evaluating new designs for culture flasks, illustrating a next step towards developing self-contained bioreactors for culturing in marine environments. Through pilot studies, an underwater system was developed to cost effectively produce cultures that yielded 200 mg of nystatin per deployment. Overall, this study demonstrates the potential for the practical culturing of microbes in a marine environment and provides an important next step for the fledgling field of molecular mariculture.

## 1. Introduction

With dwindling access to wild stocks and high demand for marine products, it is no surprise that marine aquaculture has become the largest animal-food production sector, with over 17% of the global yield attributed [1]. In 2012, almost half (49%) of the fish and seafood consumed was obtained from aquatic farming and over 18% obtained from engineered marine culturing systems commonly known as mariculture [1]. While well recognized for animal and plant production [2], the microbial aspects of mariculture have been limited to understanding the microbiome within these artificial environments [3]. In these studies, the focus has been both on monitoring [4] as well as understanding how to address bacterial infections [5], with *Vibrio sp.* [6] playing a leading role. For the latter, the application of antibiotics within these mariculture systems has become a common solution to regulate microbial outbreaks [7].

Only recently have probiotic strategies emerged as a sustainable vector to regulate microbial populations, with *Phaeobacter inhibens* providing an excellent example [8]. Here, the addition of live cultures of *P. inhibens* within feedstocks, was shown to not induce major imbalances in the microbiome, but rather, target closely related taxa associated with microbial infections. This concept of microbial augmentation has been suggested as an alternative to large-scale antibiotic use. Remarkably, although both antibiotic and probiotic strategies have been tested or are used in the field, the vectors used in both approaches are prepared in terrestrial laboratories. Ironically, many of these antibiotics and their microbial producers already exist in marine ecosystems. For example, neomycin, a critically important aminoglycoside used in fish mariculture, is produced by culturing a marine microbe, *Streptomyces fradiae* CGMCC 4.7387 [9], yet most of the neomycin used was produced in a laboratory. This disconnect has failed to be addressed both in terms of its ecological and economical perspectives due in part to a lack in methods for culturing marine microbes at sea (the term sea is used to represent marine ecosystems both within costal an open waters).

In 2014, we reported an artificial marine sponge as a tool to harvest, collect, and culture natural product producing microbes at sea [10]. This study demonstrated how concepts of biologically-inspired engineering could be used to create a self-contained microecosystem for natural product lead discovery (see excellent examples directed at soil microbes by Lewis and Epstein [11,12]), demonstrated by the isolation of the actin-targeting jasplakinolides. Soon after this discovery, it became clear that technological advances were needed to encourage the production of natural products at sea.

Realizing that there was a clear lack of studies exploring the production of natural products at sea, an effort was launched to benchmark future designs. A prototype was developed with the goal of providing an easily deployable system that would present minimal ecological impact. To guide this effort, six criteria were identified, as follows:

- Efficient: the system should be able to culture at > 10 mg L^−1^;
- Cost effective: the system should be designed to culture at < $50 USD L^−1^;
- Energy free: no external power should be required;
- Deployable: the system should require minimal training and effort for deployment;
- Contained: no microbe should be released into the environment; and
- Invisible: the system should not disrupt its proximal ecosystem.

Aquatic environments comprise approximately 71% of Earth’s surface. With the human population predicted to reach 10 billion in 35 years (UN prediction by 2056), our access to land based resources will become increasingly complicated. Over the last decade, profound efforts have been dedicated to explore the production of food, establishing safe and effective methods for marine aquaculture. Currently, nearly all molecules are produced in a terrestrial environment, often using simulated aquatic systems. This report describes a cost effective approach to culture a well-known antifungal polyketide in a marine environment; a critical next step in understanding the potential of the field of molecular mariculture (molecular production within marine environments).

## 2. Results

**Figure 1:**
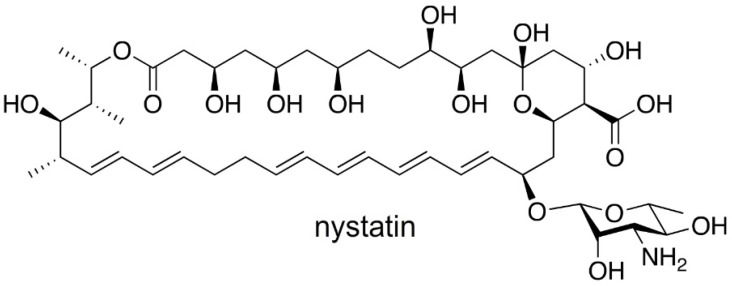
Structure of the antifungal agent, nystatin. Like amphotericin B and natamycin, nystatin can act as an ionophore. While the exact mode and mechanisms of its antifungal action remain contested, nystatin has been shown to bind to sterols within fungal cell membranes. When present in sufficient concentrations, it forms pores in the membrane that lead to K+ leakage, acidification, and cell death.

Discovered in 1950 by Brown and Hazen, nystatin [13] (Figure 1), a polyketide-derived polyene macrolide bearing a critical D-deoxymycosamine, was identified from a soil actinobacterium, *Streptomyces noursei.* Recent genome mining efforts have identified related synthases, such as the reedsmycin synthase, from marine strains (*Streptomyces youssoufiensis* OUC6819) [14], suggesting that these polyenes are also present in marine environments. Like amphotericin and natamycin [15], nystatin is an ionophore that binds to egosterol on fungal wall membranes forming pores that enable K^+^ ion leakage, ultimately leading to fungal cell death. Along with other modes of antifungal action, nystatin demonstrates a high selectivity index towards fungal strains, and hence has become one of the most commonly prescribed medicines in the United States (230th in 2017). Nystatin, sold under the brand name Mycostatin among others, is used to treat Candida infections of the skin including diaper rash, thrush, esophageal candidiasis, and vaginal yeast infections [16,17]. It may also be used to prevent candidiasis in patients who are at high risk [18]. Based on its historical and clinical importance, the production of nystatin from *Streptomyces noursei* ATCC 11,455 was selected as the target for these studies.

### 2.1. Design and Prototyping

Targeting a 2 L culturing system, our studies began by carefully examining flask design. Given the complexities of currents and tides, our goal was to identify a flask that was easy to deploy and could adapt ocean dynamics to encourage mixing (many microbial cultures require aeration via shaking or stirring for efficient production). After evaluating both engineered designs as well as commercially available 2 L containers, the devices could be classified into three different types.

**Figure 2:**
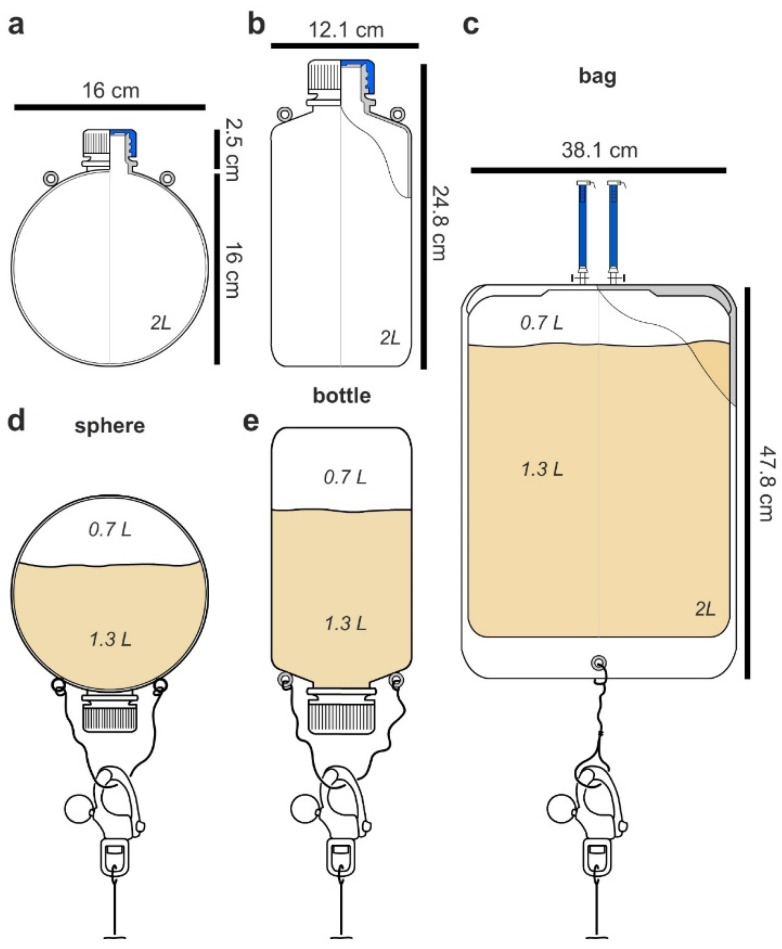
Flasks. Three different flask types were explored: (**a**) a LPDE spherical flask; (**b**) a LPDE bottle (comparable to Nalgene narrow mouth LDPE bottle 2202-0005); and (**c**) a sterile bag (Flexboy FFB102704). The spherical flasks and bottles was custom manufactured with a 16 cm diameter and 1.5 mm thickness and contained 2 eyelets for attachment. An identical capping system was used for the bottles and spherical flasks (see specifications for Nalgene 2202-0005). Each unit was deployed by attachment to a 5/16” galvanized eye and eye swivel (Pro-LifT 3478). Attachments were made using 1/8′ (3.2 mm) type 316 stainless steel 1 × 19 wire (Alps, Chicago, IL, USA). The flasks were mounted using a Stainless Steel Standard S-Bail Snap Shackle (RONSTAN–2 11/16” L) to ease in deployment.

**Figure 3:**
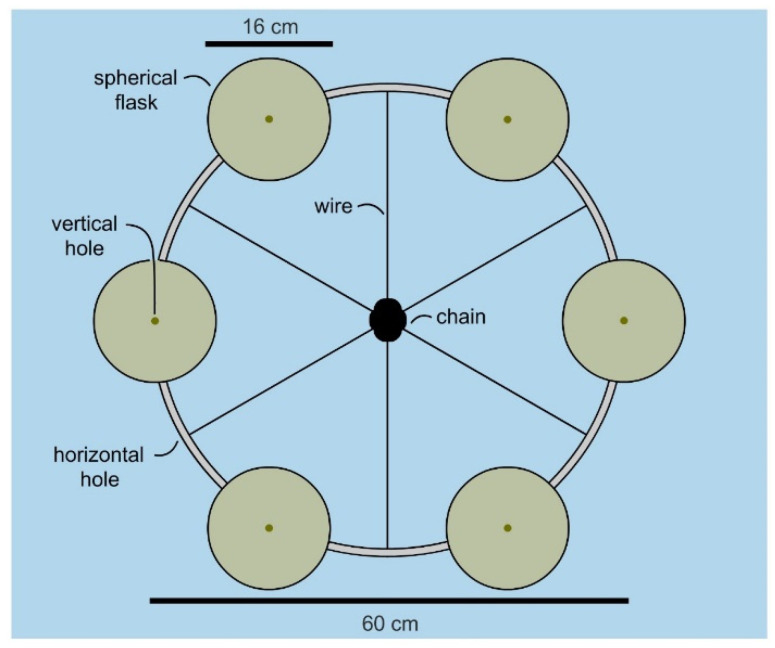
Arial (top view) of a six-flask manifold. Twelve 8 mm holes were drilled into a 60 cm stainless steel mast hoop (24” hoop, Sailrite 11730). The first 6 were drilled vertically (top to the bottom) at 60° intervals. The second set of 6 were drilled horizontally at 60° intervals offset 30° from the vertical holes. Here, alternating horizontal and vertical holes allowed attachment of the flasks (vertical) and mooring chain (horizontal). Using stainless steel wire rope (1/8′ or 3.2 mm type 316 stainless steel 1 × 19 wire, Alps), 6 snap shackles were mounted 30 cm from the hoop and attached by a marine-grade Brazier head rivet. The system was deployed by passing each wire through a single link of mooring chain, and then riveting them when taunt with a riveting clamp (commonly used for sail rigging). This process was either conducted on a boat (prior to installation of a mooring rig) or underwater (to an existing mooring rig), typically ≤10 min. A snap clamp was used to allow practical attachment and harvesting of the flasks (see Figure 4).

**Figure 4:**
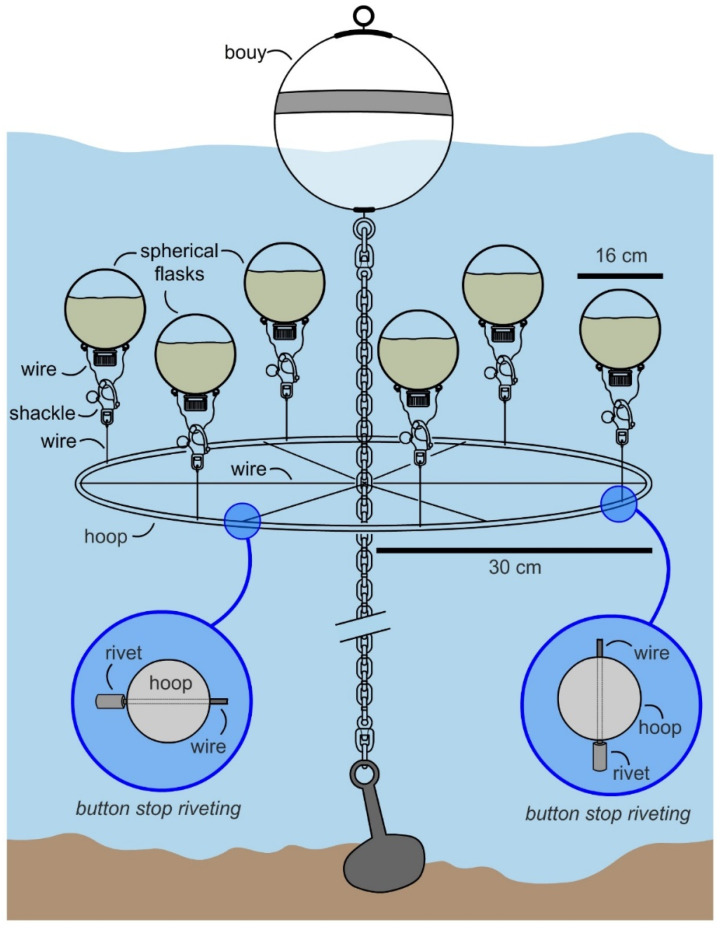
A plug-and-mariculture system. A system was designed to culture six 2 L spherical flasks. The manifold shown in Figure 3 was mounted on a mooring through a single chain link. Six flasks could be attached though shackles. Sizes are provided for the manifold and flask. Culturing was conducted at 1.3 L per flask with the remaining volume (0.7 L) charged with air.

The first or spherical design (Figure 2a), while not commercially available, offered several advantages. When floating above or underwater, it would behave like a buoy. Spherical buoys offer many advantages in preventing snags, reducing fouling, and providing minimal drag allowing them to effectively navigate currents. The second design incorporated commercially available 2 L low-density polypropylene (LDPE) laboratory bottles (Figure 2b). The use of these bottles was advantageous as it offered both low cost and availability. As shown in Figure 2a,b, eyelets were required on each flask or bottle so they could be attached to a culturing scaffold (see later discussion associated with Figure 3 and Figure 4). While these eyelets could be attached (cyanoacrylate glue, Super glue), they were optimally incorporated during the molding stage. To that end, both spherical and bottles used in this study were molded with natural translucent 100% virgin LPDE with a 38 mm closure and 1.5 mm wall thickness (reducing the commercial ease in the bottle or second type). The third or bag design (Figure 2c) was tested using commercially available culture bags (Figure 2c). Using 2 L sizes for each (Figure 2), we turned our attention to test their deployment in the field.

### 2.2. Field Testing

Each system was then tested by incubating 1.5 L of 50 µM solution of rhodamine B for the period of 1 week. Low concentrations of a fluorescent dye were used for the first deployments to ensure that the systems did not leak. Using stainless steel wire and a standard snap shackle (Figure 2), each system was attached to a mooring line (32°45′51.5″ N, 117°14′56.1″ W) at a 1 m depth. As each system had a 2 L volume, air in this void allowed each unit to float upright during its deployment. Within a few hours, it was clear that the bag system (Figure 2c) was non-ideal. First, the ports used for filling were not only cumbersome but also suggested failure. While this issue could be addressed by redesigning the bag, the movement of the bag was cumbersome and routinely wrapped itself around the mooring line. Within 6 h, it was clear that the bag type systems failed, as it would require constant monitoring and untangling. While the bottle system would be effective for short periods, the added simplicity of the spherical design enabled practical fluid dynamic calculations to further guide its deployment. Here, one could practically adjust the buoyancy of the flask by regulating the amount of media. After deployment for 1 week, the concentration of rhodamine B remained the same in both the spherical and bottle designs as determined by fluorescence analyses on a plate reader (HTS 7000, Perkin Elmer, Hong Kong), therein confirming that the first two flask types (Figure 2a,b) could be deployed without leaking their content.

Over multiple tests, inexpensive marine-grade hardware provided an effective means to attach the flask to a mooring chain as shown by the manifold in Figure 3. Incorporation of a snap shackle (a fast action fastener commonly used in sailing) allowed one to attach and retrieve flasks with minimal effort. Using commercial materials, each flask could be attached at a cost of $86 USD ($3 for wire, $56 per snap shackle and $27 per flask). While the wire was readily reused, both the shackle and flask were cleaned and reused (limit of testing 10 deployments), therein reducing the cost to ≤$10 per deployment per flask.

With a flask identified, the attention turned to developing a manifold that would allow multiple flasks to be cultured in parallel. The goal was to design a cost effective system that allowed 6 flasks to be deployed simultaneously. As shown in Figure 3, a manifold was constructed from a commercially available 24” stainless steel mast hoop (Sailrite 11730) by drilling evenly spaced holes in alternating positions (vertical or horizontal, Figure 3). Using wire rope (1/8′ or 3.2 mm type 316 stainless steel 1 × 19 wire, Alps), we were able to attach the hoop to a mooring chain by riveting (see insets in Figure 4) wire strung through the horizontal holes (Figure 3). The flasks were attached by wiring shackles on each of the six vertical holes (Figure 3). Overall, a manifold was constructed for $380 (hoop at $16, 300 cm wire rope at $8, six snap shackles at $355 ($56 each) and 12 rivets at $1) using wholesale marine tackle.

### 2.3. Deployment

In May 2017, the manifold was attached to an existing mooring (32°45′51.5″ N, 117°14′56.1″ W) at a 1.5 m depth (top of submerged flasks at ~1 m), as illustrated in Figure 4. A single manifold was prepared for this study and was operational for over 3 months with regular cleaning. After ensuring stability over 1 week, 6 flasks were prepared with 1.3 L of SAO-23 media (see Culturing in Materials and Methods) and 39 mL of inoculant was added. The flask was charged with air and capped. Each flask was then attached the manifold at 07:00 (15–20 min required for deploying 6 flasks) and allowed to culture. During this period the temperature of the water was between at 19 °C (low) at 07:00 to 23 °C (high) at 14:00. The flasks were collected after 12 h and transported to laboratory for evaluation.

## 3. Discussion

**Figure 5:**
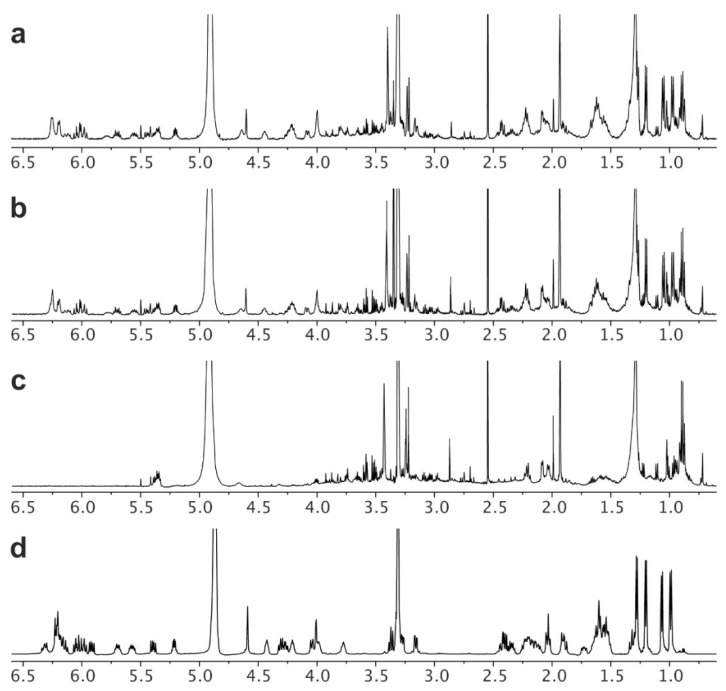
NMR analyses of maricultures. ^1^H NMR spectra (600 MHz) in CD_3_OD of: (**a**) deployment A; (**b**) deployment B; (**c**) media that was deployed under the same protocols as (**a**,**b**) without inoculation (negative control); or (**d**) a sample of purified nystatin. NMR spectra in (**a**–**c**) was taken on crude extracts prior to purification (fractionation and recrystallization). The negative control (**c**) identifies peaks in (**a**,**b**) that were derived from the media. NMR spectra are provided from 0.5 to 6.5 ppm. Additional spectral data, including further expansions of the ^1^H NMR spectra in (**a**–**c**) as well as gCOSY, NOESY, HSQC and HMBC data on purified nystatin in (**d**) have been provided in the Supplementary Material.

Nystatin was harvested from all 6 flasks (2 deployments, 12 flasks total) into a single crude nystatin product per deployment (2 crude extracts). As described in the Materials and Methods section, this began by collecting the biomass from each culture by filtration through a wire sieve. The resulting material was extracted with MeOH (see Isolation in the Materials and Methods section) to deliver a crude product. By NMR analyses, it was clear that nystatin was present in the crude extracts from deployments A (Figure 5a) and B (Figure 5b). Running the system without inoculation (a negative control) and conducting the same isolation procedure returned 0.35 g of a crude product (Figure 5c) that accounted for a majority of the impurities within these MeOH extracts (Figure 5a,b).

Next, a two-step purification and recrystallization procedure was used to obtain high-purity nystatin. First, an established solvent fractionation protocol using acetone and Et_2_O was adapted to purify the nystatin (see Purification in the Materials and Methods section). The resulting fractionated product was then recrystallized (see Recrystallization in the Materials and Methods section) to afford 204.2 mg and 208.1 mg of nystatin, from deployments A and B, respectively. NMR analysis indicated that this material was pure (Figure 5d).

A practical screening system was developed to monitor these devices using a combination of HPLC, LC–MS and/or 1.7 mm capillary cryo-NMR methods. LC–MS [19] and HPLC [20] analyses were developed at a limit of 1 ng/mL and 50 mg/mL, respectively, using established protocols. While useful, we found that 1.7 mm capillary cryo-NMR provided improved analytical evaluation. Using standards, we were able to conduct capillary NMR analysis using 30 µL samples on solutions (CD_3_OD) that contained ≥1 µg of nystatin, providing a limit of detection at 30 mg/mL. Using capillary NMR analyses, a protocol was developed using sterile swab collection system (BD 220144, BD Scientific, Waltham, MA, USA) as a means of sampling in the field. After collection and return to the laboratory, the swabs were incubated for 15 min in a 1 dram glass vial with sufficient MeOH to cover the swab (~1 mL). The swab was removed and the MeOH dried by N_2_ flow. Capillary NMR analysis was conducted on these samples by adding 50 µL to each vial and transferring 30 µL of this solution to a 1.7 mm NMR tube.

Swabbing of the outside of the cap and neck area from each of the 12 deployed flasks (6 from deployments A and B) returned NMR spectra without signal. While the rapid dilution at sea prevent this method from definitively identifying a leak, both pH and salinity testing of each flask, provided further support that the flasks did not leak. During the time of deployment, the pH and salinity of the surface water at the collection site was measured at 8.2 and 33.7 ppt. Culture broths in contrast had a pH ranging from 5.8 to 7.2 [21] and low salinity (<50 ppm). While not measured, online digital monitoring of pH and salinity of the culture broths would provide an excellent tool to precisely monitor for leakage. Here, one could envision the addition of a WiFi or Bluetooth pH or salinity meter to each flask.

## 4. Materials and Methods

**Culturing.** All flasks were sterilized by washing with absolute EtOH and/or autoclaving. The studies were conducted using published methods for culturing nystatin from *Streptomyces noursei* ATCC 11,455 [21]. Fermentations were conducted using 1.3 L of SAO-23 media (90 g L^−1^ glucose · H_2_O, 3 g L^−1^ corn flour, 7 g/L Ca_2_CO_3_, 2.5 g L^−1^ NH_4_NO_3_, 0.4 g/L MgSO_4_ · 7 H_2_O, 0.2 g/L KH_2_PO_4_, and 3 mL of trace elements. Trace element solution was prepared with 5.0 mg mL^−1^ FeSO_4_ · H_2_O, 0.39 mg mL^−1^ CuSO_4_ · H_2_O, 0.44 mg mL^−1^ ZnSO_4_ · 7 H_2_O, 0.15 mg mL^−1^ MnSO_4_ · H_2_O, 0.02 mg mL^−1^ CoCl_2_ · 6 H_2_O, 0.01 mg mL^−1^ Na_2_MoO_4_ · 2 H_2_O, and 50 mg mL^−1^ HCl. Inoculant for each fermentation was prepared at 3% volume (39 mL for each 1.3 L culture) using Tryptone Soya Broth (TBS) (Waltham, MA, USA) media with 37 g L^−1^ Oxoid 129 (ThermoFisherScientific, Waltham, MA, USA) at 28 °C in 500 mL baffled Erlenmeyer flasks with 100 mL of medium at 200 RPM for 18 h after addition of 0.2 mL of spore suspension [21]. Inoculants were transported for deployment in sterile 50 mL conical tubes. Inoculations were conducted just prior to deployment.

**Isolation.** Nystatin was isolated using published methods [22]. Briefly, cultures of *S. noursei* were filtered through a stainless steel wire strainer (5FWT6, Humbolt, Lake Forest, IL, USA) and dried by pressing with filter paper (Whatman, Maidstone, UK) until dry. All 6 cultures from each deployment (2 deployments tested) were harvested using a single filter and filtration. Crude concentrates of nystatin can be prepared by extracting the mycelial mat with MeOH. This was conducted by soaking the filter in a stainless steel tray (10 inch diameter, 3 inch height stainless steel pan used) with sufficient MeOH to cover the biomass (~700 mL) and gently shaking on a rotary platform shaker for 1 h at 23 °C so that the solution is gently passing over the steel mesh. The MeOH extract was collected, and the process was repeated 5 times and the supernatants were pooled (final volume of ~4 L).

**Purification.** Crude nystatin was obtained by fractional precipitation of the MeOH fraction (~4 L) using 20 L Nalgene polypropylene carboys (3 required, carboys were washed between steps) with EtOAc (1 L) for 30 min, collecting the supernatant, repeating this precipitation 3 times with sequential additions of EtOAc (1 L). At this point, an additional 4 L of EtOAc was added to the 8 L ~1:1 mixture of EtOAc/MeOH. The resulting precipitate was washed with 0.85% aq. NaCl (2 L), dissolved in MeOH (~2.5 L) and subjected to fractional precipitation with Et_2_O. Comparable to the prior precipitation, the 2.5 L solution in MeOH was added to a 20 L Nalgene polypropylene carboys (2 required, carboys were washed between steps) with Et_2_O (500 mL) for 30 min, collecting the supernatant, repeating this precipitation three 3 times with sequential additions of Et_2_O (650 mL). At this point, an additional 10 L of Et_2_O was added to the 5 L ~1:1 mixture of Et_2_O/MeOH and the resulting precipitate was harvested after sitting for 6 h. Over the two repetitions, this process returned 2.1 g (deployment A) and 2.4 g (deployment B) of crude product.

**Recrystallization.** Nystatin was further purified from the crude product by recrystallization [23]. A saturated solution of NaI in acetone was prepared. A 2 g portion of crude nystatin was added to 8 mL NaI saturated acetone. The crude nystatin product dissolved after stirring for 1 h. The solution was clarified by decanting the supernatant. Next, *n*-BuOH (25 mL) was added to this solution followed by deionized H_2_O (6 mL). The solution was warmed to 40 °C in a water bath and 5 mg of nystatin (Fisher Bioreagents, Waltham, MA, USA) was added to seed. Crystalline material was observed within 15 min. After 1 h, the water bath was removed and the solution was stored in the dark for 22 h. The product was collected by vacuum filtration and washed with a mixture having the same composition as the mother liquor (8 mL of NaI saturated acetone, 25 mL *n*-BuOH and 6 mL of H_2_O). Two repetitions on ~2 g of the fractionated products from deployment A and B returned 204.2 mg and 208.1 mg of crystalline nystatin, respectively.

## 5. Conclusions

Overall, this study demonstrated a method to culture microbial strains at sea as demonstrated by the production of 0.4 g of pure nystatin from cultures of *Streptomyces noursei* ATCC 11455. This study met 5 of the 6 design criteria (efficient, cost effective, energy free, deployable, contained and invisible). In terms of efficiency, this prototype can be realized at low cost. Including media ($58 per deployment at $7.5/L, pricing based on laboratory biochemical pricing) and the system ($9 per deployment), this mariculture system was able to deliver over 200 mg of nystatin over 3 days (1 day for deployment and 2 days for isolation, purification and recrystallization). Extraction of the inoculant alone would have returned quantities of <1 mg/flask, indicating that this system was viable for microbial growth and compound production. While not cost effective at this test scale (reagent-grade nystatin sells at $15/g), this study did meet the remaining criteria (energy free, deployable, contained and invisible).

While energy was required to travel to the mooring, the system was deployed and harvested by hand and did not require any energy to operate. The spherical design of the flask provided an excellent underwater buoy that encouraged mixing of the media. Here, tides and currents were used to provide continual motion. While this mimicked the aeration used in laboratory culturing, it was far less effective than that possible by shaker flasks or mechanical stirring in a bioreactor. Further engineering efforts could provide improved manifold designs (built to capture currents) and flasks (built to shake during stirring such as baffling) that would further enhance media mixing by uniting ocean currents with mechanical stirring within a flask.

Overall, the system (Figure 4) was easy to deploy, as flasks were attached to the manifold by surface diving (snorkel assisted) by means of a shackle. The system met the requirements for easy deployment as all 6 flasks were deployed within 10 min. The system was underwater and not visible from the surface and was contained (preliminary tests were established to monitor leakages). While not fully optimized, the system did deliver mg L^−1^ productivity with a yield of 26 mg L^−1^ of nystatin per deployment.

This study was conducted using a terrestrial organism (*Streptomyces noursei* ATCC 11455). One can immediately see the potential of this approach for culturing marine-derived and marine-obligate microbes. As marine microbes are already adjusted to growth at sea, one can envision a system wherein microbial culturing and surrounding environments are developed symbiotically, wherein flasks and manifolds provide a direct mimicry of the natural microbiological growth. While recognized as a resource for drug discovery [24,25,26,27], this study now suggests that marine ecosystems may one day provide an environment for drug development and production. Given the expanse of mariculture and our understanding of the critical ecological factors in its proper implementation, this study suggests a new potential for microbial culturing at sea.

## Data Availability

Not applicable.

## Supplementary Materials

The following are available online, Copies of 1D and 2D NMR spectra have been provided. Figure S1. Expansion of ^1^H NMR spectra provided within the manuscript.; Figure S2. Expansion of ^1^H NMR spectra provided within the manuscript.; Figure S3. Expansion of ^1^H NMR spectra provided within the manuscript.; Figure S4. Expansion of ^1^H NMR spectra provided within the manuscript.; Figure S5. Expansion of ^1^H NMR spectra purified nystatin from deployments A (top) and B (bottom); Figure S6. ^13^C NMR spectrum and spectral expansions of purified nystatin from deployment A.; Figure S7. ^1^H, ^1^H-gCOSY spectra of purified nystatin from deployment A.; Figure S8. ^1^H, ^13^C-HSQC spectra of purified nystatin from deployment A.; Figure S9. ^1^H, ^13^C-HMBC spectra of purified nystatin from deployment A.
