# Supplementary material for: Accessing Nystatin through Mariculture
Source: Molecules. 2021 Dec 17;26(24):7649. doi: 10.3390/molecules26247649 (PMC8708966; doi:10.3390/molecules26247649)

Supplementary Materials

Accessing Nystatin through Mariculture

James J. La Clair [1]*

[1] Xenobe Research Institute, P.O. Box 3052, San Diego, CA 92163-1052, USA; i@xenobe.org

* Correspondence: i@xenobe.org (J. J. L.)

Figure S1. Expansion of ^{1}H NMR spectra provided within the manuscript.

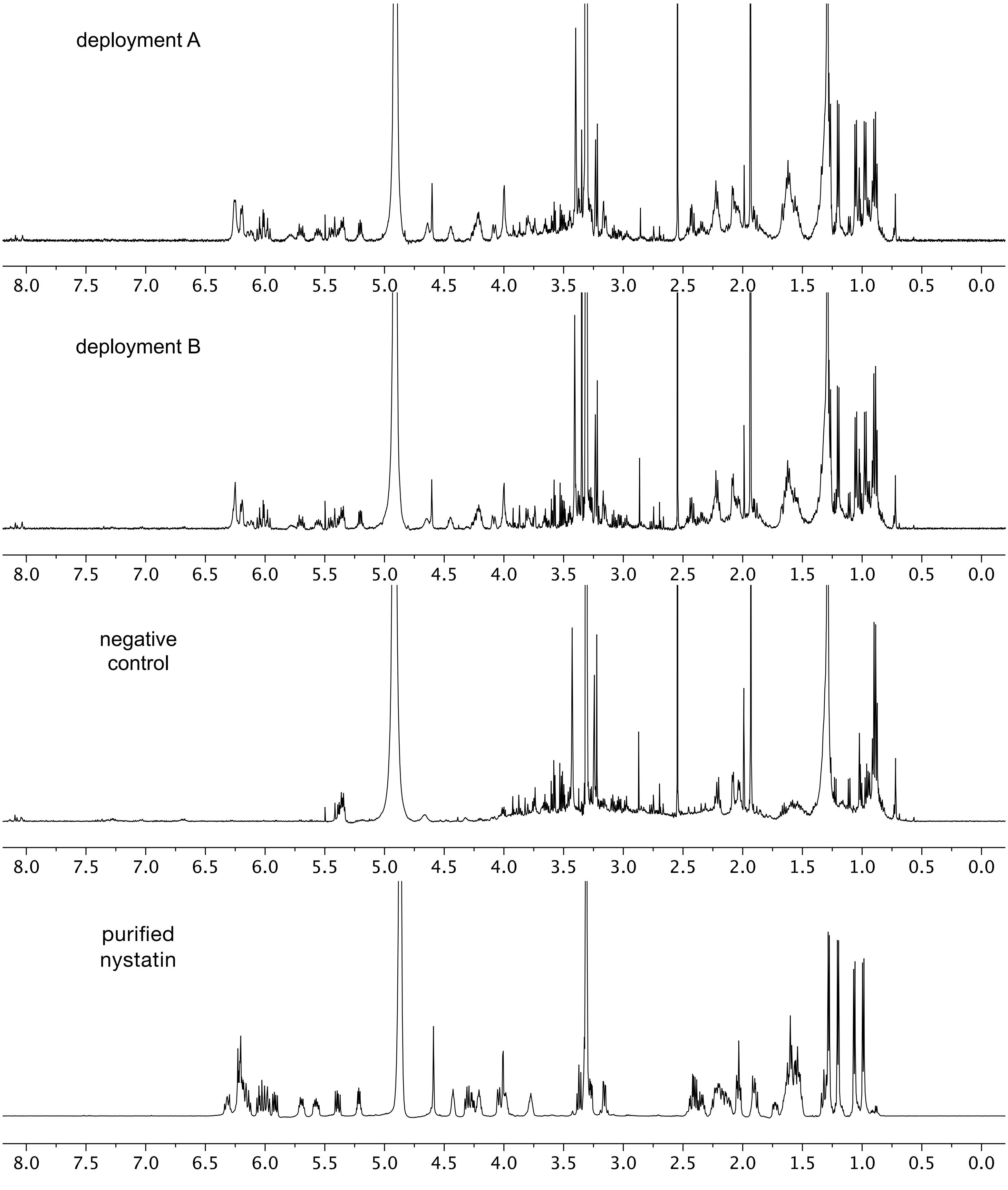

Figure S2. Expansion of ^{1}H NMR spectra provided within the manuscript.

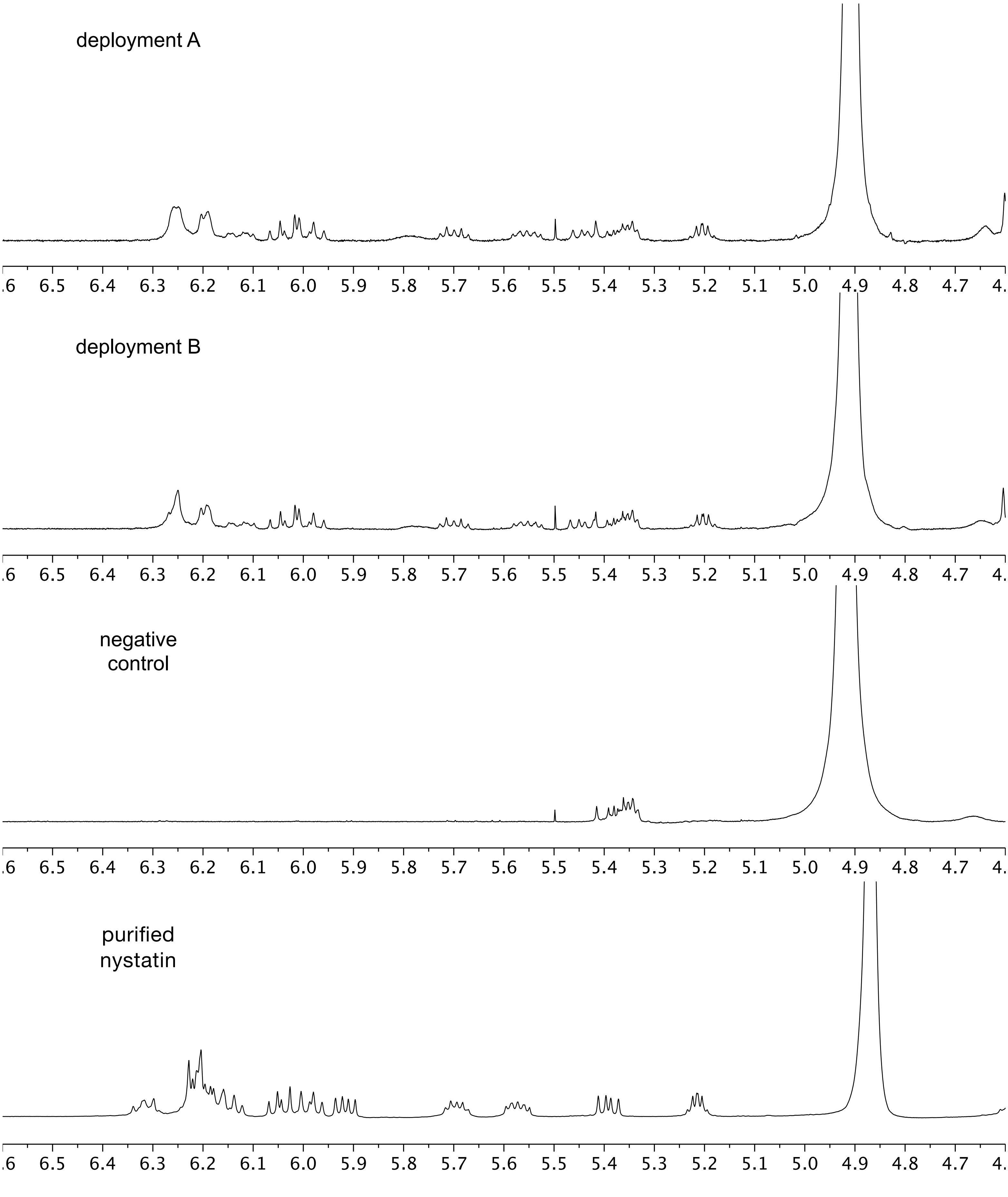

Figure S3. Expansion of ^{1}H NMR spectra provided within the manuscript.

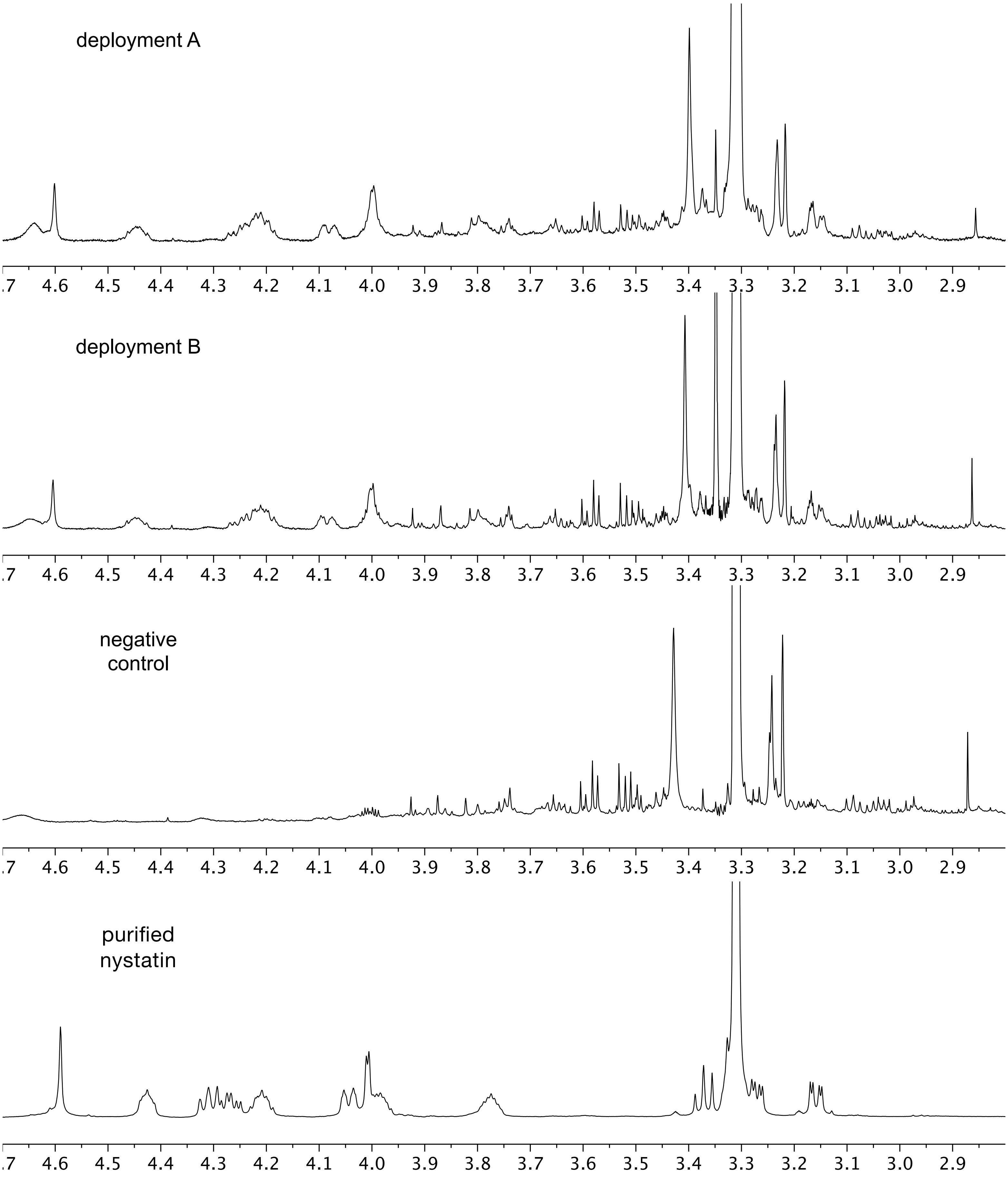

Figure S4. Expansion of ^{1}H NMR spectra provided within the manuscript.

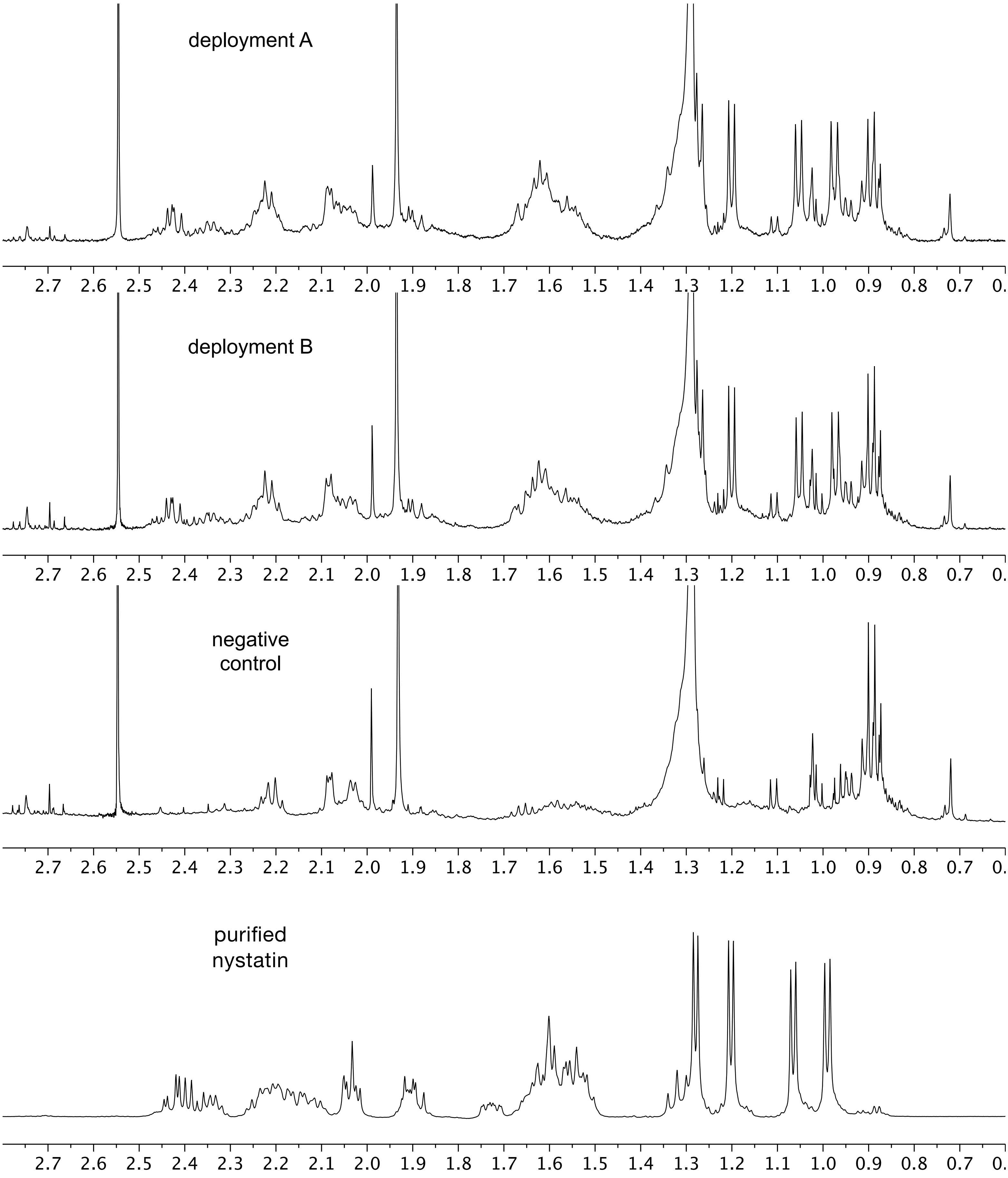

Figure S5. Expansion of ^{1}H NMR spectra purified nystatin from deployments A (top) and B (bottom)

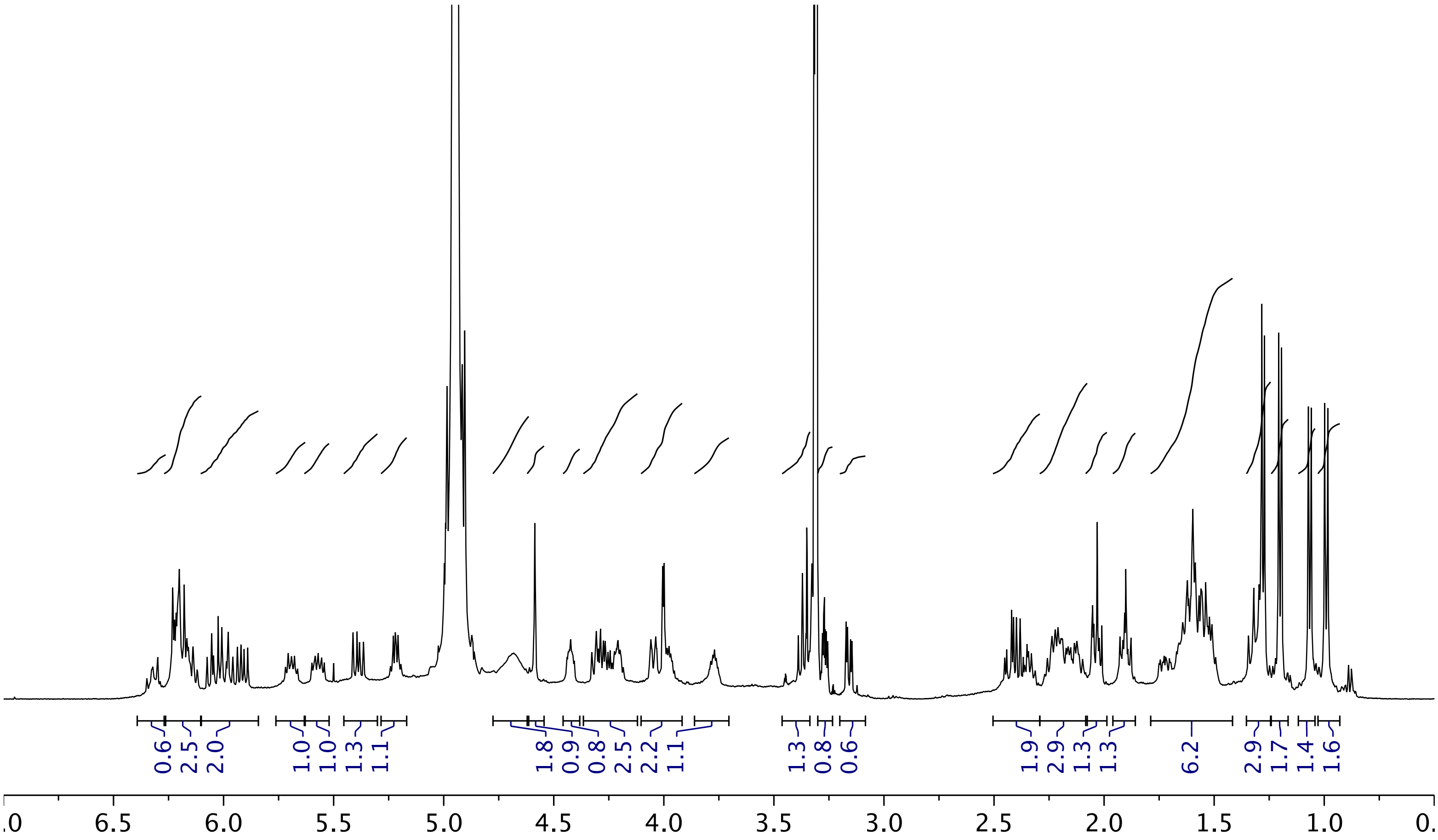

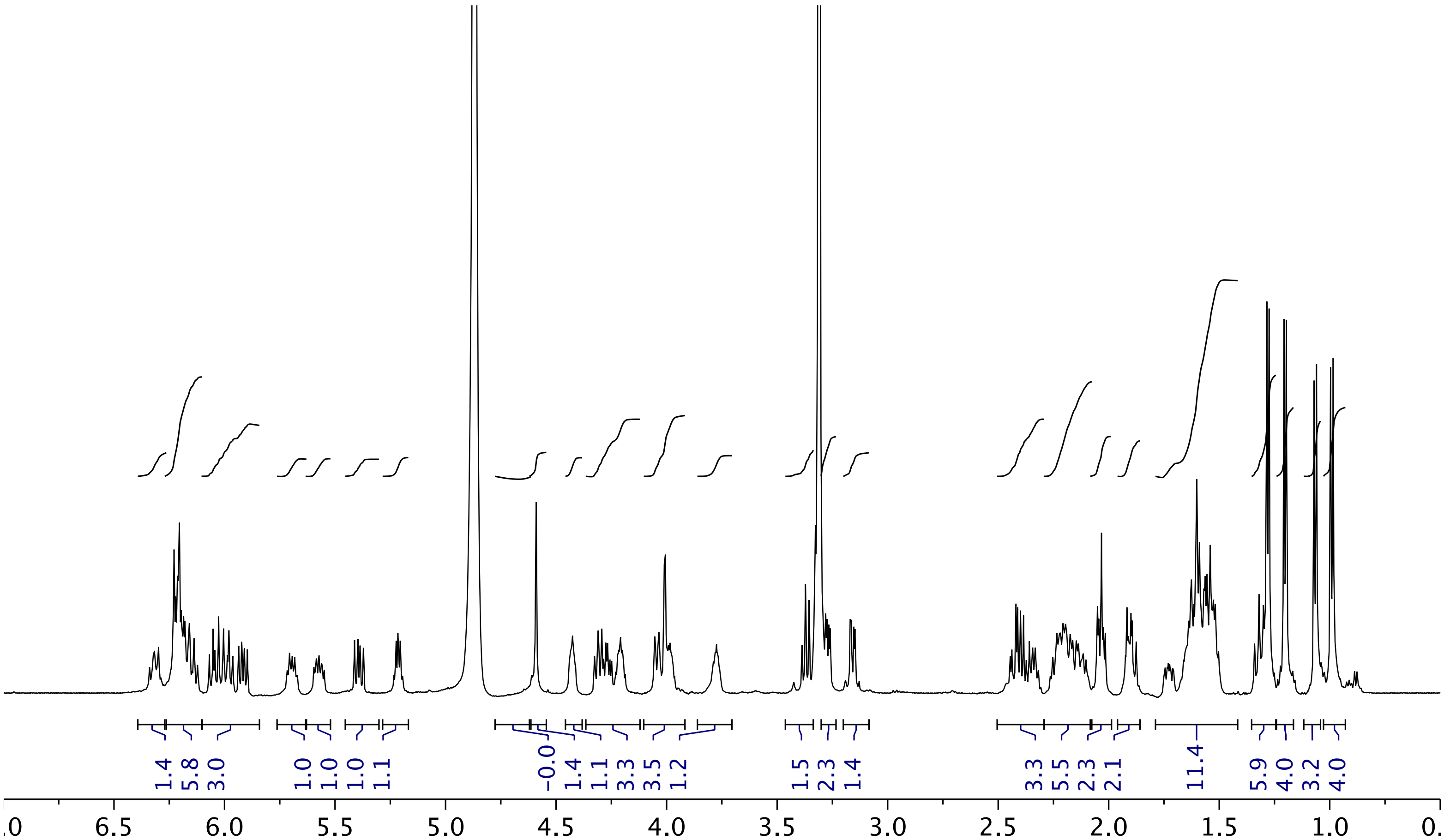

Figure S6. ^{13}C NMR spectrum and spectral expansions of purified nystatin from deployment A

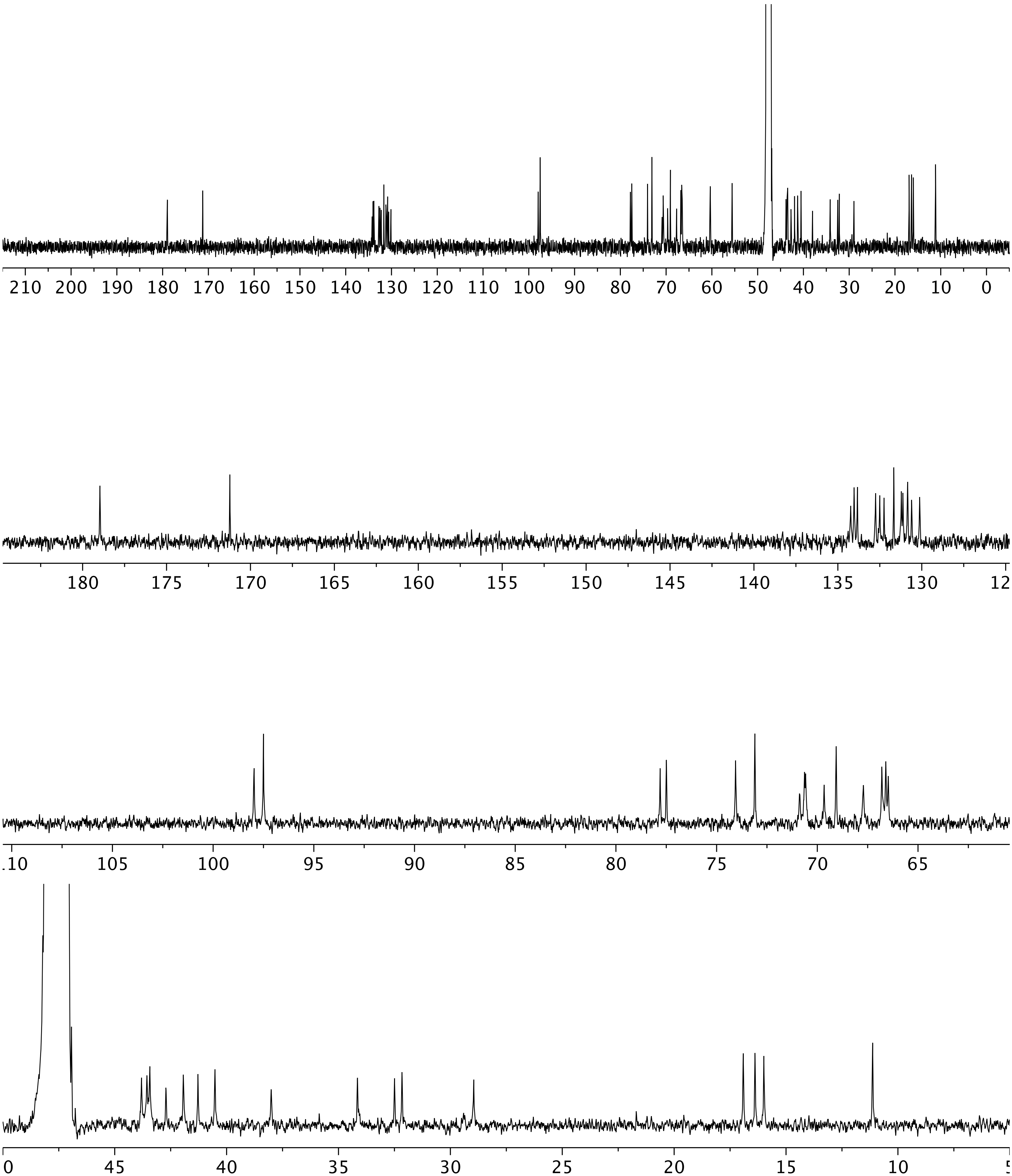

Figure S7. $^{1}H,^{1}H$-gCOSY spectra of purified nystatin from deployment A

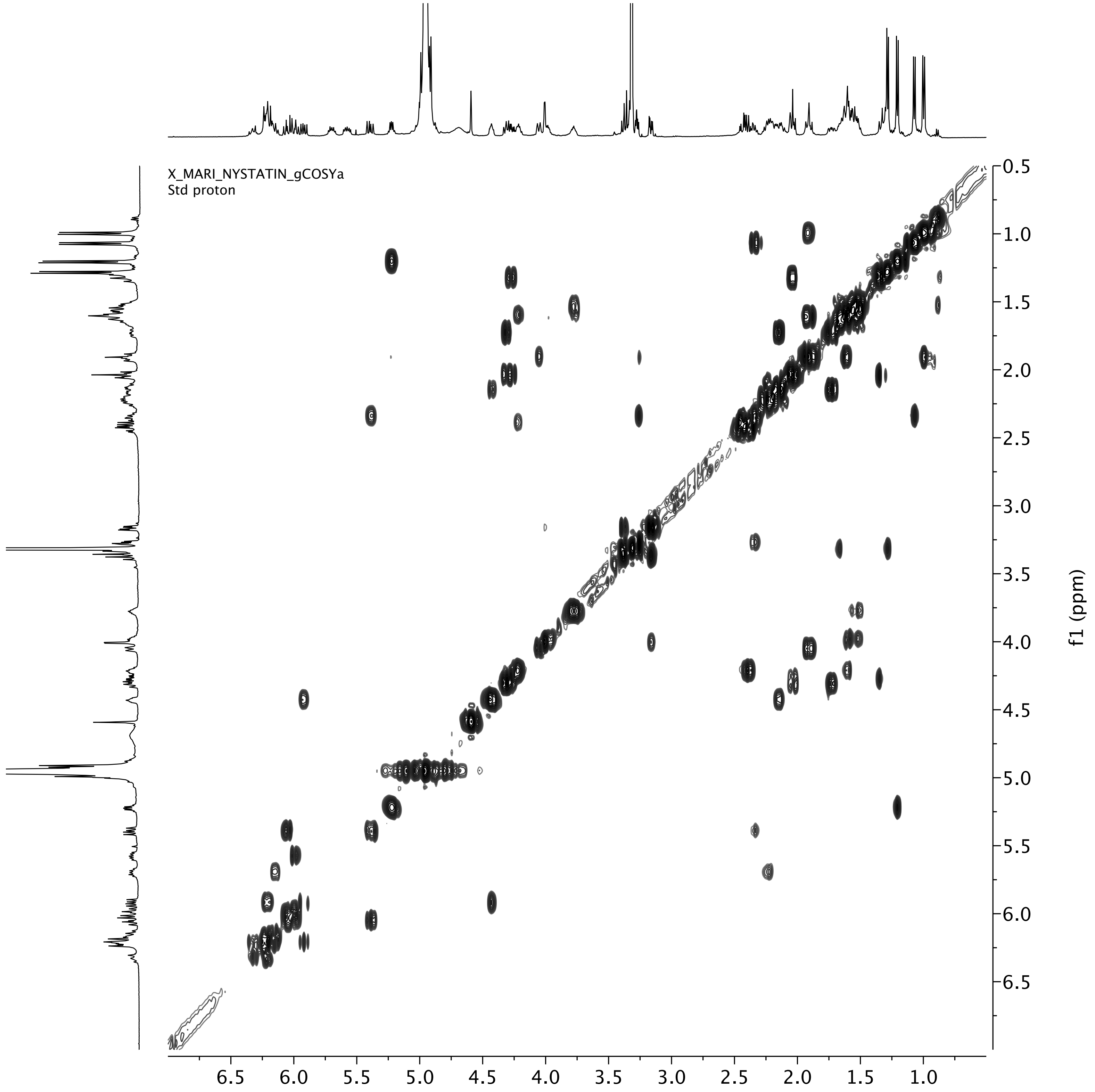

Figure S8. ^{1}H,^{13}C-HSQC spectra of purified nystatin from deployment A

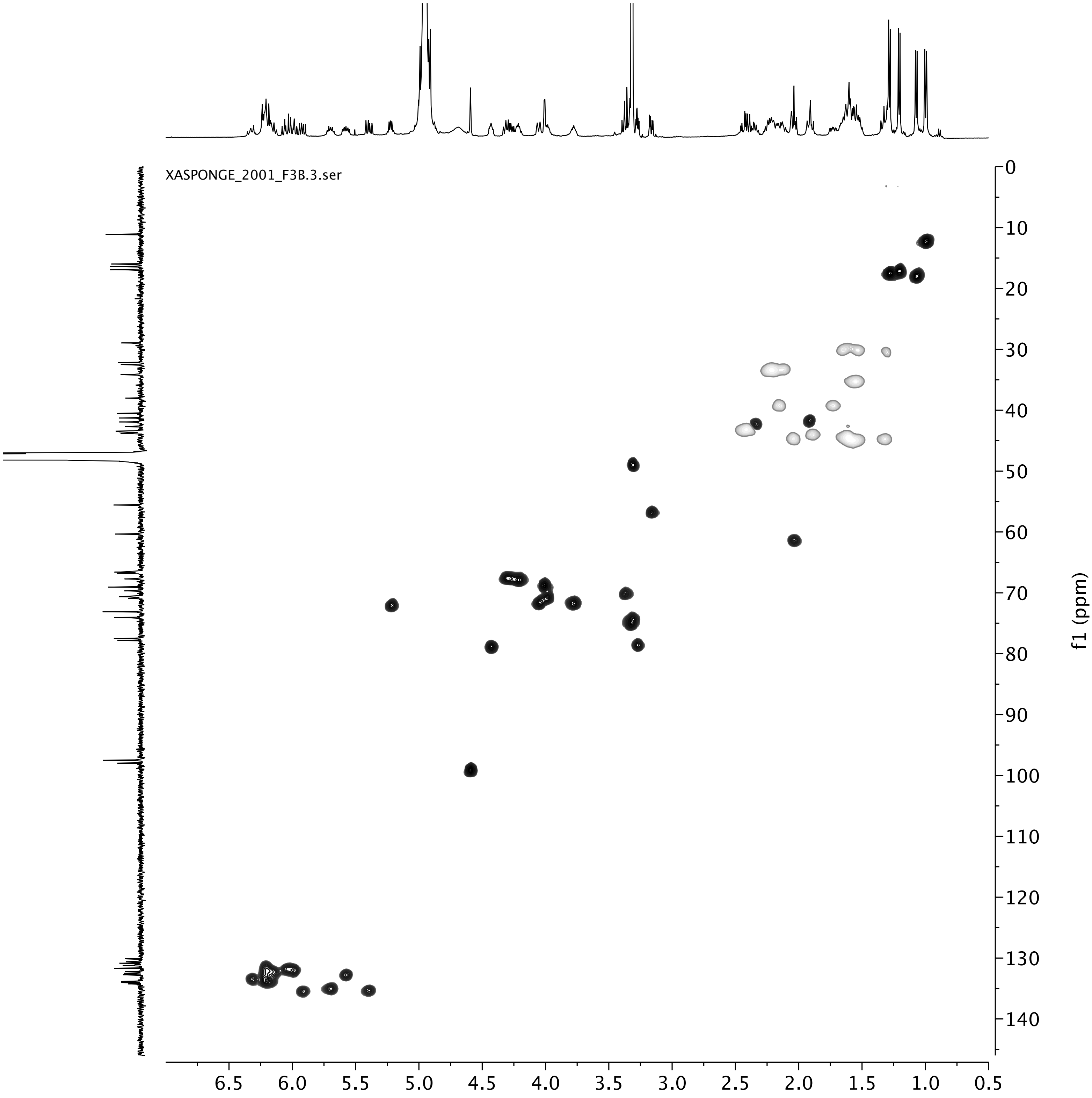

Figure S9. ^{1}H,^{13}C-HMBC spectra of purified nystatin from deployment A

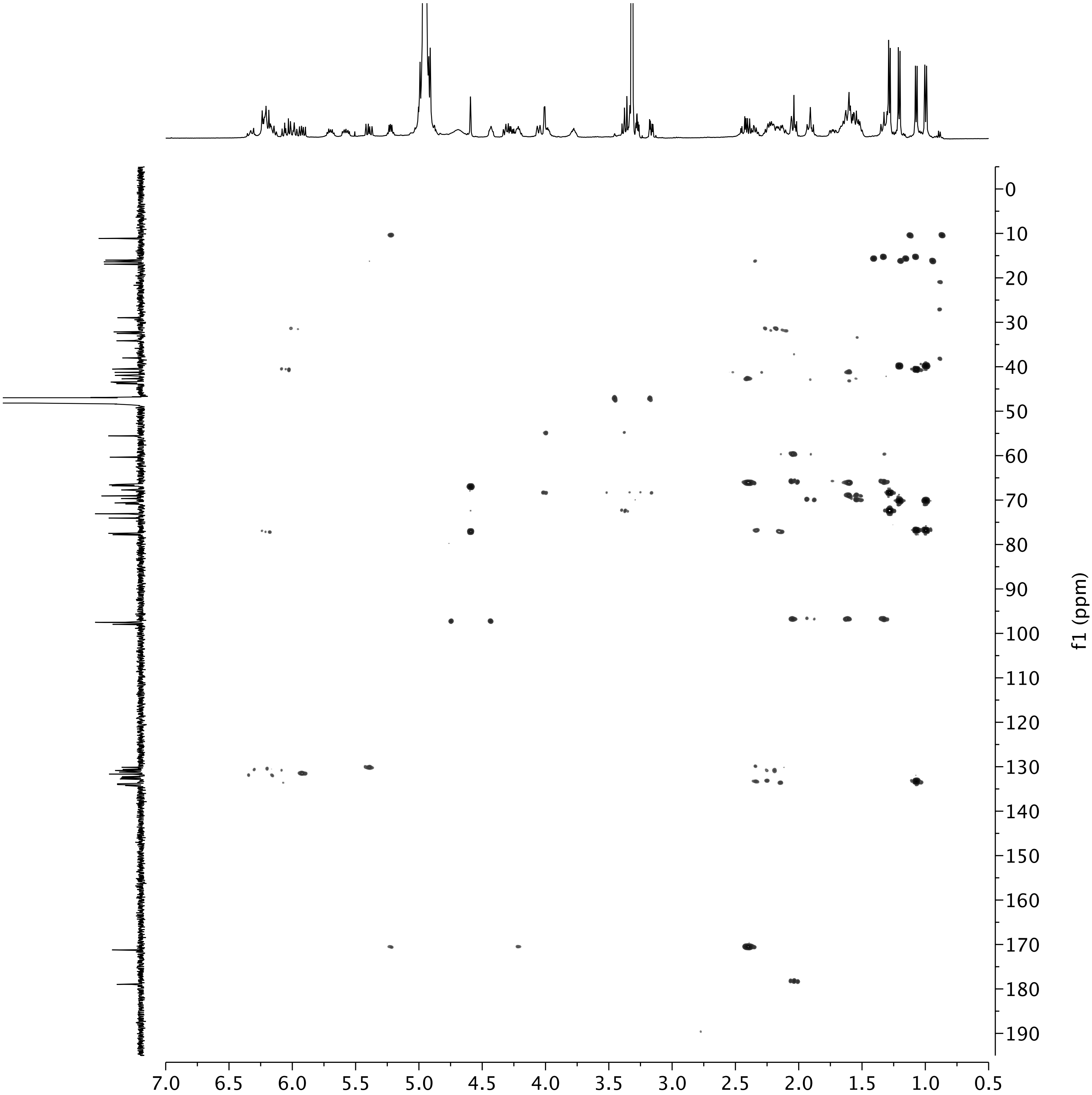

Supplement: Supplementary file 1 [file molecules-26-07649-s001.zip › molecules-1467829-supplementary.pdf]
